# Reliability and Validity of Feminist Identity Composite in Chinese Women

**DOI:** 10.3389/fpsyg.2019.02842

**Published:** 2019-12-20

**Authors:** Yingjiang Liu, Yong Zheng

**Affiliations:** Key Laboratory of Cognition and Personality (Ministry of Education), Southwest University, Chongqing, China

**Keywords:** feminist identity, validity, reliability, Chinese women, genderism

## Abstract

This study evaluated the psychometric properties of a Chinese form of the Feminist Identity Composite (FIC). A total of 2,334 Chinese adult women completed the scale for this study. In study 1 (*n* = 875), exploratory analysis indicated six factors, Passive Acceptance, Revelation, Embeddedness/Emanation, Active Commitment, and Synthesis, the latter of which was divided into two subscales (Femininity Synthesis and Autonomous Synthesis). In study 2 (*n* = 810), confirmatory factor analysis was conducted with a different sample to examine the construct validity of the model obtained in study 1. In study 3 (*n* = 649), the convergent validity of the FIC was examined via their relationship with willingness to engage in feminist behaviors and attitude toward trans-people. The results indicated that a new measurement with solid conceptual and psychometrically solid properties needs to be developed to assess Chinese women’s feminist identity.

## Introduction

The Feminist Identity Composite (FIC; Fischer et al., 2000) is a self-report measure of beliefs associated with feminism and a kind of collective or social feminist identity. The FIC has been used in feminist identity research, and since its original development in 2000, over 20 published studies have used the FIC as a measure to assess feminist beliefs and identification. A particular strength of the FIC is that it includes five sequential stages—more specifically, five feminist ideologies—of the feminist identity development process.

The FIC, which employs the best items from the Feminist Identity Scale (FIS) (Rickard, 1989) and Feminist Identity Development Scale (FIDS) (Bargad and Hyde, 1991), is based on feminist identity development theory (Downing and Roush, 1985). According to experience in psychological practice, and with reference to Black Identity Development Theory (Cross, 1971), Downing and Roush (1985) suggested that feminist identity develops through five stages: (1) Passive Acceptance (PA), in which women systematically accept traditional gender roles and their subordinate status due to lack of awareness of gender discrimination; (2) Revelation (REV), in which women have undergone some crisis and contradiction that has caused them to break away from traditional gender roles, they also feel deceived, angry, and guilty about the past; (3) Embeddedness/Emanation (EE), in which women begin to form emotional connection with other women and groups, although the relationship with male relatives sometimes hinders this process (Moradi et al., 2002); (4) Synthesis (SYN), in which women reconstruct their self-conception to include the positive characteristics of women and their own unique attributes; and (5) Active Commitment (AC), in which the most important thing for woman is to apply their feminism to positive social movements and strive to achieve gender equality. In short, Downing and Roush’s theory conceptualized a developmental process of how women may acquire and maintain a positive feminist identity (Fischer et al., 2000).

While the FIC has been validated with different samples, Chinese women have not been included. Hence, in the present study, we aimed to examine the psychometric properties of the FIC, in a sample of Chinese women from diverse demographic and geographic backgrounds so as to provide evidence of the FIC’s cross-cultural validity or lack thereof.

### New Generation of Chinese Feminists

The voice of feminism in China is not as silent as we may think. On Valentine’s Day, 2012, three young women wore wedding dresses smeared with ‘blood stains’ in order to oppose domestic violence, in the first public appearance of young Chinese feminists. The same year, a few young women who self-identified as ‘young feminist activists’ launched a social movement named ‘Occupy the Men’s Toilet,’ which marked the birth of a new generation of Chinese feminists (Li and Li, 2017). Since then, similar feminist social movements have emerged in China for different social causes, such as opposition to sexual harassment, domestic abuse, and job discrimination. Unlike the older feminist generation of the 1980 and 1990s, who have stayed closely aligned with the government (Wang, 2018), the new generation is more internationally aligned and consistent with Western feminists. First, the new generation is characterized by ‘optimism, sincerity, social progressiveness, and devotion to community,’ and connected by ‘personalized’ digital networks (Wang and Driscoll, 2019). What is more, the new generation of feminists have more diversified social and organizational backgrounds, as well as lifestyles (Li and Li, 2017). Particularly, the new generation has the participation of LGBTQ feminists, who openly defy heterosexual normativity, and a strong focus on body politics (Wang and Driscoll, 2019). Above all, they are willing to use ‘feminism’ to identify their movements and self-identified as feminists.

### Support

Is it reasonable to expect that the FIC could capture Chinese women’s feminist identity? To answer this question, it is important to understand the development of feminism in China.

The development of feminism in China has gone through four stages. The first wave of Chinese feminism took place since the May Fourth Movement in 1919. As leaders of the movement, traditional male intellectuals proposed feminism with individual liberation and women’s emancipation as its key characteristics. Foot binding, access to education for women, women joining the workforce, and female participation in government were the main topics of their discussion.

The second wave occurred after the founding of the People’s Republic of China in 1949 (Shen, 2016). The Chinese Communist Party (CCP) defined gender equality as equality between men and women, and gender differences were written off (Yang and Yan, 2017). Women’s rights to education, employment, and participation in politics largely materialized.

The third wave began in the 1980s and ended in the 1990s. During this stage, the reform and opening up policy was initiated, and it brought about drastic changes on the fronts of both gender and class. Women assumed an inferior position in the labor market, and images of feminized women began to flood popular culture, thereby reinvigorating traditional gender norms. Meanwhile, Western feminist theories were introduced to China by some female intellectuals. People began to learn about women’s rights and gender ideology (Shen, 2016). The rise of women’s self-awareness and the formation of feminist groups were the two main events of this stage.

Considered to have begun around the turn of the century, the fourth wave of Chinese feminism does not have a clear time point. However, it has notable characteristics which differentiate it from the first three waves and make it more in line with the international feminist movement. First, the fourth wave is led by the grassroots with diverse backgrounds; in contrast, the first three waves were led by Chinese male intellectuals, the government, or female academics. Second, the fourth wave is an independent movement to achieve gender equality. The first and second waves, meanwhile, were significant parts of China’s nationalist movement and socialist revolution, and did not greatly promote the development of gender equality because women’s liberation was attached to nation-building endeavors (Huang, 2016). Moreover, the fourth wave feminists have organized collective actions, such as *The Vagina Monologues*, to gain influence and achieve their goals, similar to their western counterparts. In addition, they focus on not only education and jobs for women but also sexual misconduct and LGBTQ issues, which are also topics addressed by the fourth wave of the international feminist movement.

Some research evidence has also indicated that feminist identity among Chinese samples is not very dissimilar from their Western counterparts. First, genderism and female’s rights is an international issue and women in different countries are struggling for it. Cheung and Halpern (2010) found that Chinese and American women leaders converge in terms of interweaving work and family roles on their way to the top, and they attributed such similarities to women sharing what we call ‘the culture of gender,’ which exerts stronger impact on women than do socio-political ideologies. Second, some similarity of gender related ideologies exist between Western countries and China. Li et al. (2017) compared tolerance for intimate partner violence with college students from China and the US and the consistent pattern of tolerance for intimate partner violence influenced by attitudes toward gender roles.

Above all, Western feminist ideologies have had an important effect on Chinese feminist development. Particularly, after The Fourth World Conference on Women, which was held in Beijing in September 1995, women’s civic associations came to life (Han, 2018), and the young feminist generation has been more in line with international feminism. Hence, it is reasonable to expect that the FIC, to some extent, could capture Chinese women’s feminist identity.

### Opposition

Nevertheless, although some similarities have emerged, it cannot be denied that the different cultural and social backgrounds may lead to weak validity and reliability of the FIC in the context of China.

Drawing from multiracial feminist theory, the experiences of all individuals are shaped by the multiple social statuses they occupy (Harnois and Ifatunji, 2011). While emphasizing the intersectional nature of hierarchies at all levels of social life (Baca Zinn and Thornton Dill, 1996), multiracial feminist theory hypothesizes that feminist identification, the salience of feminism in women’s lives and what women understand feminism to be, may differ across racial and ethnic groups (Harnois, 2005; Robnett and Anderson, 2017).

Compared to the West where the FIC originated, Chinese culture mainly has three notable characteristics. First, China is one of the most typical collectivist countries. Traditional Chinese culture emphasizes that the essence of human beings is ‘the ensemble of social relations,’ which is defined as ‘Ren’ in Confucianism (Sun, 2015). What is more, prioritizing harmony is also one of the core values of Chinese collectivist culture. Such doctrine makes any behaviors that undermine interpersonal relationships or social stability illegal. Second, in China, women’s obedience to fatherhood has a profound cultural foundation which has existed for 1000s of years. Deriving from the traditional ethics and patriarchal feudalism, the female culture indoctrinated women with ‘man is superior to woman,’ ‘three obedience’ (obey her father before marriage, and her husband during married life and her sons in widowhood) and ‘four virtues’ (fidelity, physical charm, propriety in speech and efficiency in needle work). All the ethics defined women’s the ideal gender role as the virtuous wife, filial piety woman and good mother. Third, feminism in China has its own emphasis which is distinct from that in Western countries. Zhu and Li (2015) found that the feminist movement in China emphasizes equal division of family and work roles between men and women, while in the US, the emphasis is on equal rights between men and women in terms of employment, education, and politics. This difference in framework is due to the unique feminist organization and development process in China. Although the fourth wave of Chinese feminism has been more in line with international feminism, the first three waves, which are largely distinct from Western feminism, still exert a considerable influence on how women regard gender equality.

In addition to the difference in culture, Chinese feminists face a more severe and stifling social context. ‘Feminism’ has become a politically sensitive word, and feminist organizations have generated a backlash from the authorities (Andersen, 2018). For example, on the eve of International Women’s Day in 2015, five feminists were detained for planning to distribute anti-sexual harassment stickers (Xiao, 2019). Further, Feminist Voices, a feminist group’s social media account with 180,000 followers, was shut down, and the official reason for the closure was vague (Andersen, 2018). In addition to the pressure from the authorities, misogyny has increasingly become a problem faced by Chinese feminists when engaging in online spaces (Han, 2018). Although there is no decisive evidence to determine which country exhibits greater misogyny in cyberspace, online misogyny has been entwined with negative connotations of feminism, which may distort individuals’ perceptions of feminism.

Based on the above discussion of the reasons for and against the applicability of the FIC for Chinese women, the present study aims to solve this problem by reanalyzing the psychometric properties of the FIC following the method of the original scale development.

#### Psychometric Properties of the FIC

Recently, some studies have been done on the psychometric justification for the FIC. For reliability, the Cronbach’s alpha of five subscales was acceptable (see Table 1). However, samples in the extant studies were mostly white women with a limited age spectrum, particularly college students. Blue and Berkel (2010) employed the FIC with 100 black students, in which a relatively low Cronbach’s alpha for the five subscales, particular PA, indicated that African American women’s experiences may not be adequately captured by the FIC, nor by Downing and Roush’s theory.

**TABLE 1:** The Cronbach’s α of five subscales and the demographic information of participants in the previous studies.

**References**	**Size**	**Age range**	**Age (*M* ± *SD*)**	**Race**	**PA**	**REV**	**EE**	**SYN**	**AC**
Fischer et al., 2000	191	18 to 34	19.4 ± 2.4	90%White	0.75	0.80	0.84	0.68	0.77
Moradi and Subich, 2002	240	16 to 67	30.2 ± 12.7	79%White	0.74	0.76	0.84	0.73	0.77
Szymanski, 2004	227	18 to 72	38.3 ± 11.3	85%White	0.67	0.6	0.78	0.48	0.81
Fischer and Good, 2004	191	—	19.4 ± 2.4	90%White	0.74	0.75	0.86	0.71	0.81
Sabik and Tylka, 2006	256	17 to 48	19.8 ± 4.2	77%White	0.80	0.80	0.91	0.75	0.72
Yakushko, 2007	691	18 to 83	40.0	89%White	0.79	0.79	0.90	0.68	0.85
Yoder et al., 2007a	125	18 to 61	19 ± 6.1	79%White	0.71 to 0.81	0.60 to 0.78	0.80 to 0.87	0.66 to 0.77	0.79 to 0.89
	103	19 to 48	21.0 ± 4.6	89%White					
Yoder et al., 2007b	165	18 to 63	19.0 ± 7.0	73%White	0.76	0.78	0.79	0.65	0.79
Peterson et al., 2008	276	—	20.6 ± 3.4	70%White	0.78	0.83	0.76	0.72	0.79
Blue and Berkel, 2010	100	18 to 30	—	African	0.62	0.76	0.75	0.83	0.78
Yoder et al., 2011	220	18 to 39	18.5 ± 2.3	85%White	0.68	0.86	0.85	0.76	0.83
Backus and Mahalik, 2011	183	18 to 22	18.9 ± 0.9	81%White	0.75	0.79	0.87	0.71	0.83
Yoder et al., 2012	255	18 to 39	19.0 ± 2.4	85%White	0.74	0.86	0.85	0.76	0.83
Erchull and Liss, 2013	326	18 to 30	23.1 ± 3.0	86%White	0.73	0.64	0.72	0.62	0.80
Kucharska, 2015	273	20 to 65	35.4 ± 11.2	Polish	0.88	0.93	0.90	0.92	0.83
DeBlaere et al., 2017	544	18 to 78	35.0 ± 14.5	72%White	0.72	0.71/0.73	0.86	0.77	0.86
Luu and Inman, 2018	235	—	—	80%White	0.74	0.74	0.88	0.60	0.87

*PA, Passive Acceptance; REV, Revelation; EE, Embeddedness-Emanation; SYN, Synthesis; AC, Active Commitment.*

Regarding construct validity, Fischer et al. (2000) conducted confirmatory factor analysis (CFA) and their good model fit indexes affirmed the five-factor structure of the FIC as corresponding to the five stages of feminist identity. In addition, Moradi and Subich (2002) also examined the structural validity of the FIC and found that all item parcels significantly loaded on assigned factors ranged from 0.27 (S) to 0.84 (EE). However, most of fit index values (except for RMSEA) approached but did not reach recommended cut-offs (Moradi and Subich, 2002). In order to provide the psychometric evidence of the FIC with samples from diverse women, DeBlaere et al. (2017) employed the FIC with two subsamples of sexual minority women, and found that while the dimensionality of feminist identity existed in sexual minority women, there were still some distinctions among women of different sexual orientations. The evidence discussed above, on the one hand, has provided some support for the psychometric properties of the FIC, while on the other hand, has yielded an urgency to re-examine the cross-cultural validity of the FIC with diverse samples, which can help determine the differences in feminist identity between Chinese and Western women, as well as provide references for the revision of the FIC.

Concerning convergent validity, the FIC has been found to correlate with other feminist-related ideologies and criteria. For example, Yoder et al. (2011) reported correlations between the FIC subscale scores and affective attitudes toward the feminist movement and feminism (ranging from 0.19 to 0.50), as well as the endorsement of egalitarianism (ranging from 0.23 to 0.37). Further evidence suggests significant correlations between the FIC subscales scores and willingness to engage in feminist activities (ranging from 0.25 to 0.48, Szymanski, 2004), which indicated that women with strong feminist identity tend to contribute to collective endeavor. In addition, the development of the FIC occurred during the third wave of the feminist movement, during which, efforts were made to break the boundaries and conceptions of gender. The rights of transgender and gender non-conforming people have been supported by feminists. Platt and Szoka, in press found that endorsement of feminist beliefs is an independent inverse predictor of transphobia. Further, DeBlaere et al. (2017) also reported moderate relationships (ranging from 0.22 to 0.47) between the FIC subscale scores and heterosexism.

#### The Current Study

The current study attempts to examine the psychometric properties of the FIC for women in a culture, particularly the culture of mainland China, which is different from that of Western countries. First, Study 1 assessed the dimensionality of the FIC based on an exploratory factor analysis (EFA) to find the underlying structure of the FIC with a Chinese sample. Subsequently, in Study 2, the factorial validity of the FIC scale scores was examined with a different sample of Chinese women using CFA, a powerful data analytic technique to evaluate *a priori* factor structure (Floyd and Widaman, 1995). Two models were evaluated, including a correlated six-factor model and a five-factor, second-order model. The FIC’s psychometric properties were further investigated via the relationship between the subscales and internal consistency coefficients. Finally, in Study 3, we investigated the convergent validity of the FIC scores among the Chinese sample through the correlation of the FIC with willingness to engage in feminist behaviors and with genderism and transphobia.

## Study 1: Exploratory Factor Analysis

We used a Chinese sample to explore whether the item composition of the FIC subscales had changed. First, based on the reviewed studies, it was found that the new generation of feminism in China does share some feminist ideologies, as reflected in the FIC, with Western feminists, such as a collectivist orientation. In addition, the factor pattern of the FIC, which has been examined in a diverse sample (although Asians were not included), has been found to be convincing. Therefore, we hypothesized that (H1a) the item scores of the Chinese version of the FIC will have a five-factor structure, namely PA, REV, SYN, EE, and AC. Second, because of the poor performance of SYN in previous research, such as having the lowest Cronbach’s alpha coefficients (Erchull et al., 2009), and because the items in SYN are rooted in individualism (Downing and Roush, 1985; Erchull et al., 2009), we further hypothesized that (H1b) except SYN, the other four subscale items will load primarily on the factor they belong to.

### Materials and Methods

#### Measure

##### Feminist Identity Composite (FIC)

The FIC is a 33-item Likert-type scale, on which items are rated from (1) *strongly disagree* to (5) *strongly agree* (Fischer et al., 2000), with higher scores means indicating more consistency with a particular stage. It consists of five subscales, Passive Acceptance (PA, e.g., ‘I like being a traditional female’); Revelation (REV, e.g., ‘Gradually, I am beginning to see just how sexist society really is’); Embeddedness/Emanation (EE, e.g., ‘I am very interested in women writers’); Synthesis (SYN, e.g., ‘I have incorporated what is female and feminine into my own unique personality’); and Active Commitment (AC, e.g., ‘I want to work to improve women’s status’). Fischer et al. (2000) reported that the Cronbach’s alphas of five subscales are 0.75 for PA, 0.80 for REV, 0.84 for EE, 0.68 for SYN, 0.77 for AC. The psychometric properties of the FIC used in the present study are discussed later.

In the current study, the FIC was translated into Chinese by two bilingual (Chinese and English) graduate psychology students. The several cycles of back translation were conducted by a bilingual (Chinese and English) English major. Then, we invited four Chinese college students from Southwest University to check the Chinese-translated FIC and present recommendations about necessary cultural adjustments. Finally, two bilingual (Chinese and English) personality psychologists and four bilingual (Chinese and English) advanced psychological doctoral students compared the Chinese and English versions of the FIC to evaluate their conceptual and linguistic equivalence on a four-point scale from (1) *totally different* to (4) *identical* (Jeanrie and Bertrand, 1999). The average conceptual equivalence and linguistic equivalence scores of all items ranged from 3.3 to 4.0 or 3.2 to 4.0, respectively.

#### Procedure

The questionnaire was administered via a professional survey website, *Wenjuanxing*^1^, during September 2018 in mainland China. We shared the link of the questionnaire on popular social networking platforms, such as Weibo, WeChat, and Baidu Tieba. Chinese women over the age of 18 years were allowed to complete the questionnaire. The ethics research committee of Southwest University approved this study. The informed consent form for recruitment (the data were only used for scientific study and kept anonymous) was presented before the questionnaire began. Participants first completed a demographic questionnaire and then the FIC. No compensation was directly given to participants, except, after completing the survey, participants were entered into a lottery draw with a prize worth from 10 RMB to 200 RMB.

#### Participants

We obtained 884 completed questionnaires. For the purpose of the study, we excluded juveniles (age < 18; *n* = 9). The final sample consisted of 875 Chinese women from 28 provinces/regions of mainland China. The age of the participants ranged from 18 to 64 with a mean of 26.89 years (*SD* = 6.81). Most respondents had a full-time job (*n* = 583, 66.6%), while a small number of respondents were full-time students (*n* = 219, 25.0%), and fewer were employed part-time (*n* = 60, 6.9%) or unemployed (*n* = 13, 1.4%). Regarding education, 652 (74.5%) had a college education, 66 (7.5%) had a postgraduate education or higher, 121 (13.8%) had a junior college education, and 36 (4.2%) had high school education or less. Concerning monthly salaries, 305 (34.9%) participants earned 3,000–6,000 RMB per month, 211 respondents earned 6,000–10,000 RMB per month (24.1%), 272 respondents earned less than 3,000 RMB per month (31.1%), and 87 earned more than 10,000 RMB (9.9%).

### Results

We conducted EFA on 33 items with SPSS Version 23.0 to examine the structure validity of the FIC with a Chinese sample. The participant (875) to variable (33) ratio exceeded the recommended ratio for EFA of 10 to 1 (Cattell, 1978). Fabrigar et al. (1999) suggested that an adequate sample size is influenced by the extent to which factors are overdetermined and the level of the communalities of the measured variables. A higher participant-to-variable ratio (30:1) was therefore considered. Measures of skewness and kurtosis (skewness from −1.672 to 1.061; kurtosis from −1.126 to 3.636) demonstrated that all 33 items met the criteria for univariate normality (i.e., skewness < 3, kurtosis < 10) (Weston and Gore, 2006). No multivariate outlier cases were identified based on Mahalanobis distances significant at *p* < 0.001.

In EFA, Principal Axis Factoring with a Promax oblique rotation technique (kappa value = 4) was employed to detect the number of latent factors in the FIC. The Promax oblique rotation assumes that the factors could be related to one another. Bartlett’s test of sphericity, χ^2^(528) = 5597.834, *p* < 0.001, and the Kaiser-Meyer-Olkin value (0.825) indicated that the data were appropriate for factor analysis. The eigenvalues greater-than-one rule (Kaiser, 1960) and a scree plot (Cattell, 1966) were considered for determining the number of factors to retain. Beyond that, parallel analysis (Horn, 1965) was also used to decide the number of factors. Parallel analysis using Monte Carlo simulations was based on comparison of the extracted eigenvalues from the present sample data to those that might be expected from random data (Fabrigar et al., 1999). Calculating from 1,000 generated data sets with 875 cases and 33 variables, the parallel analysis indicated that a six-factor solution would be better, since the seventh eigenvalue obtained using principal axis factoring extraction (eigenvalues 4.80, 2.69, 2.38, 1.86, 1.37, 1.22, and 1.11) failed to exceed the seventh from the parallel analysis (eigenvalues 1.38, 1.33, 1.30, 1.27, 1.24, 1.21, and 1.19).

The criteria for item retention were as follows: (1) items loaded less than 0.32 were screened (Worthington and Whittaker, 2006), and (2) items displaying strong cross-loadings were deleted from the scale (0.32 or greater on a second factor; Tabachnick and Fidell, 2007). Following this procedure, six factors with 29 items were produced. Item PA3 (‘I don’t see much point in questioning the general expectation that men should be masculine and women should be feminine’), SYN5 (‘As I have grown in my beliefs I have realized that it is more important to value women as individuals than as members of a larger group of women’), REV3 (‘Gradually, I am beginning to see just how sexist society really is’), and AC8 (‘I owe it not only to women but to all people to work for greater opportunity and equality for all’) had failed to load on any factor, these items were discarded. The SYN subscale was divided into two parts. We called these two factors Feminine Synthesis (f-SYN), which contains two items (‘I feel like I have blended my *female attributes* with my unique personal qualities’ and ‘I have incorporated what is *female and feminine* into my own unique personality’) and Autonomous Synthesis (a-SYN), which also contains two items (‘I am proud to be a *competent* woman’ and ‘I enjoy the pride and self-assurance that comes from being a *strong* female’). f-SYN refers to the degree of integrating feminine qualities with personal attributes into a positive self-concept (Downing and Roush, 1985). a-SYN refers to integrating the independent-personality quality into a positive self-concept.

All six factors accounted for 34.21% of the total extracted variance. Factor loadings and the variance explained by each factor in the six-factor solution of the FIC are reported in Table 2. According to Tabachnick and Fidell (2007), most of the items’ loading on their factors meet the criteria of 0.45 (fair), while only three items’ loading meets the criteria of poor (0.32). The items has been shown in the Supplementary Material.

**TABLE 2:** Exploratory factor analysis six-factor solution of the feminist identity composite.

**Items**	**Factor**						***h*^2^**	**Item-total *r***
	**1**	**2**	**3**	**4**	**5**	**6**		
AC4	**0.631**	0.137	–0.038	0.068	0.035	–0.124	0.43	0.67***
AC2	**0.576**	–0.151	0.002	–0.068	–0.062	–0.014	0.28	0.56***
AC3	**0.537**	0.017	0.018	0.037	0.037	–0.085	0.29	0.62***
AC6	**0.499**	–0.028	–0.044	–0.008	0.006	0.051	0.25	0.59***
AC7	**0.461**	–0.047	–0.026	0.071	–0.109	0.211	0.27	0.55***
AC9	**0.423**	0.010	0.004	0.090	0.172	–0.072	0.25	0.58***
AC5	**0.421**	0.060	0.059	–0.071	0.012	0.104	0.30	0.57***
AC1	**0.413**	0.048	0.045	–0.066	–0.045	0.191	0.31	0.60***
REV6	–0.007	**0.675**	0.018	–0.106	0.049	–0.026	0.49	0.71***
REV5	–0.005	**0.595**	0.003	0.056	–0.056	0.117	0.36	0.65***
REV7	0.112	**0.531**	–0.007	–0.050	0.024	–0.096	0.34	0.64***
REV1	–0.054	**0.510**	–0.021	0.075	–0.032	0.008	0.24	0.59***
REV2	0.031	**0.480**	0.039	–0.071	–0.081	–0.058	0.25	0.60***
REV8	–0.131	**0.456**	0.008	–0.038	0.151	–0.035	0.22	0.56***
REV4	0.026	**0.437**	–0.009	0.146	–0.137	0.056	0.21	0.54***
EE3	–0.024	–0.071	**0.885**	0.003	0.014	–0.037	0.73	0.85***
EE1	0.053	0.024	**0.725**	0.090	–0.074	0.049	0.56	0.81***
EE2	–0.057	0.064	**0.721**	0.011	0.017	–0.074	0.50	0.79***
EE4	0.121	0.043	**0.333**	–0.177	0.106	0.092	0.30	0.64***
PA2	–0.106	0.107	0.002	**0.581**	0.001	–0.057	0.39	0.64^∗∗^
PA4	0.075	–0.020	–0.025	**0.561**	–0.053	–0.087	0.34	0.65***
PA6	0.045	0.015	0.013	**0.545**	–0.013	0.011	0.29	0.63***
PA5	–0.084	0.026	0.052	**0.512**	0.036	0.216	0.26	0.61***
PA1	–0.018	0.032	–0.030	**0.465**	0.086	–0.040	0.24	0.62***
PA7	0.095	–0.154	0.078	**0.463**	0.060	–0.022	0.24	0.60***
SYN1	–0.059	–0.008	0.020	0.019	**0.805**	0.042	0.63	0.87***
SYN2	0.089	–0.064	–0.034	0.056	**0.607**	0.061	0.43	0.87***
SYN3	0.081	–0.066	–0.008	–0.019	0.040	**0.490**	0.29	0.73***
SYN4	0.117	0.111	–0.086	0.002	0.172	**0.345**	0.25	0.86***
Eigenvalues	4.55	2.55	2.30	1.85	1.33	1.07		
% of Variance	13.55	6.39	5.63	4.76	2.64	1.24		

Retained item loadings are indicated in boldface. PA, Passive Acceptance; REV, Revelation; EE, Embeddedness/Emanation; f-SYN, Femininity Synthesis; a-SYN, Autonomous Synthesis; AC, Active Commitment. ***p < 0.001; ^∗∗^*p* < 0.01.

### Discussion

Study 1 was designed to examine the underlying structure of the FIC with a Chinese sample which was excluded from previous studies. As hypothesized, the results revealed a component composition similar to the original FIC subscales. Specifically, most items in PA, REV, EE, and AC loaded on their hypothesized factors. The results suggest that the feminist ideologies of Western feminists are shared by women in mainland China. Noteworthily, two items were dropped due to failure to load on any factors. We attribute the failure of PA3 to state-prescribed gender sameness which has a lasting impact on people’s gender ideology. Further, we ascribe the failure of SYN5 to culture clashes. Living in a society that emphasizes social embeddedness and loyalty to in-groups (Grossmann and Na, 2014), women in mainland China may prefer to evaluate individuals as group members, even women who embrace feminist identities. In addition, item REV3 and AC8 independently made up a new factor in the current sample. These two items failed to load on their original factors, and it was difficult to endow the new factor with theoretical significance, indicating that the items represent expressions of feminist identity that do not fit well for Chinese women. Therefore, we chose to drop these items.

On the other hand, the SYN subscale was divided into two subscales, each of which contained two items from the original subscale, indicating that the positive aspects of being a woman—particularly, feminine and competent—assumed in the SYN subscale are not a homogeneous structure for Chinese women. Finally, we obtained a six-factor solution, which is contrary to our original hypothesis.

## Study 2: Confirmatory Factor Analysis

**FIGURE 1:**
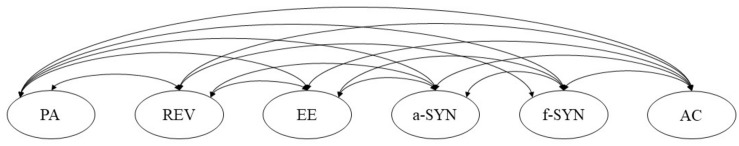
The hypothetical correlated six-factor model.

**FIGURE 2:**
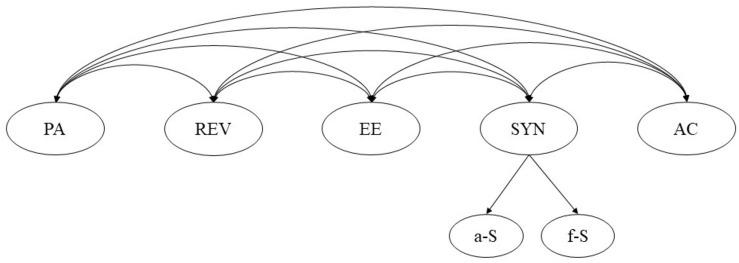
The hypothetical five-factor, second-order model.

Study 2 aimed to reconfirm the current structure obtained in Study 1. We further examined the six-factor structure of the FIC via CFAs in a different sample of Chinese women. There are two alternative approaches to represent the structure of a measure hypothesized to include several highly related dimensions: the five-factor, second-order model (see Figure 1) and the correlated six-factor model (see Figure 2). The two models differ in conceptualization of multidimensionality.

The a-SYN subscale (e.g., ‘I am proud to be a *competent* woman’) measures women’s perception of being called to enact patriarchal notions of strength, such as being aggressive, rational, and bodily strength (Connell and Pearse, 2015), while the f-SYN subscale (e.g., ‘I have incorporated what is female and *feminine* into my own unique personality’) measures women’s perception of femininity, such as career, motherhood, and beauty (Lane et al., 2016). However, for Chinese women, the figures of feminine women and strong women seem to be incompatible. The deep-rooted traditional Confucian culture defines femininity as weak and obedient, having little to do with the attributes of independence, intellect, and confidence (Ghate, 2018). Additionally, such incompatibility also stemmed from attempts by state-prescribed feminism in China to promote equality in the realm of ‘labor’ by asexualizing the subjectivity of women (Ghate, 2018), thereby undervaluing femininity. It is thus reasonable to hypothesize that (H2a) the correlated six-factor model, which treats f-SYN and a-SYN as different but related dimensions, would fit the data better.

In addition, we also examined the reliability of the revised Chinese version of the FIC by estimating the Cronbach’s alpha coefficients, item-total correlation, and correlations between the subscales. As previous studies have found that traditional gender roles are negatively related to indicators of feminist ideologies, such as female labor force participation (Chen and Ge, 2018) and disabusing son preference (Li and Jiang, 2019), we hypothesized that (H2b) PA will negatively correlate with the other four subscale scores, except f-SYN. This is because, as mentioned above, femininity assessed by f-SYN is also emphasized in traditional gender roles.

### Materials and Methods

#### Measure

##### Feminist Identity Composite

This was the same as in Study 1.

#### Procedure

The questionnaire was conducted via a professional survey website, *Wenjuanxing*^2^, during October 2018 in mainland China. The ethics research committee of Southwest University approved this study. The participants in Study 2 were recruited in the same way as in Study 1.

#### Participants

We obtained 821 completed questionnaires. For the purpose of the study, we excluded juveniles (age < 18; *n* = 11). The final sample consisted of 810 Chinese women from 28 provinces/regions of mainland China. The age of the participants ranged from 18 to 69 with a mean of 27.38 years (*SD* = 7.09). Most respondents had a full-time job (*n* = 545, 67.3%), while a small number of respondents were full-time students (*n* = 201, 24.8%), and fewer were employed part-time (*n* = 47, 5.8%) or unemployed (*n* = 17, 2.1%). Regarding education, 587 (72.5%) had a college education, 61 (7.5%) had a postgraduate education or higher, 120 (14.8%) had a junior college education, and 42 (5.2%) had high school education or less. Concerning monthly salary, 276 (34.1%) participants earned 3,000–6,000 RMB per month, 200 respondents earned 6,000–10,000 RMB per month (24.7%), 245 respondents earned less than 3,000 RMB per month (30.2%), and 89 earned more than 10,000 RMB (11.0%).

### Results

#### Confirmatory Factor Analysis

In this section, we conducted CFAs with Mplus Version 7.0. The sample size (*n* = 810) was adequate for CFA (Quintana and Maxwell, 1999). Measures of skewness and kurtosis had been conducted prior to the CFA. The results (skewness from −1.641 to 1.064; kurtosis from −1.187 to 3.860) demonstrated that all items’ distributions met the criteria for univariate normality (i.e., skewness < 3, kurtosis < 10; Weston and Gore, 2006). No multivariate outlier cases were discovered based on Mahalanobis distances significant at *p* < 0.001. Five primary fit indices were used to test model fit: chi-square statistic (*χ^2^*/*df*), Comparative Fit Index (CFI), root-mean-square error of approximation (RMSEA), and standardized root-mean-square residual (SRMR), with acceptable model fit indicated by a CFI ≥ 0.90. Further, 1 < χ^2^/*df* < 3, RMSEA < 0.06, and SRMR < 0.08 are generally considered a good fit (Bentler, 1992; Browne and Cudeck, 1992; Worthington and Whittaker, 2006).

For the correlated six-factor model, all items moderately-to-highly loaded on the intended latent factors at a significance level of 0.05; standardized factor loadings ranged from 0.36 to 0.68 for PA; from 0.38 to 0.65 for REV; from 0.56 to 0.84 for EE; from 0.65 to 0.74 for f-SYN, from 0.51 to 0.62 for a-SYN, and from 0.48 to 0.58 for AC. The model fit index met the conventionally accepted cut-offs. χ^2^(362) = 766.41, *p* < 0.001; CFI = 0.91; SRMR = 0.046; RMSEA = 0.037, 90% confidence interval (CI) = [0.033, 0.041]. We also examined five-factor, second-order model, the model fit index were χ^2^(365) = 829.209, *p* < 0.001; CFI = 0.90; SRMR = 0.050; RMSEA = 0.040, 90% CI = [0.036, 0.043]. The correlated six-factor model fit the present data better.

#### Reliability Estimates

The internal consistency of the revised FIC was examined again. The Cronbach’s alphas of subscales were 0.67 for PA, 0.71 for REV, 0.79 for EE, and 0.75 for AC. As the f-SYN and a-SYN subscales only contain two items each, we used the correlation coefficient to estimate reliability. In particular, the correlation coefficient between the two items in f-SYN was 0.49 (*p* < 0.001) and that in a-SYN was 0.32 (*p* < 0.001). The Cronbach’s alpha of the overall revised FIC (29 items) was 0.75. The correlations between the subscales and the item-total correlation were also re-examined. As reported in Table 3, in the revised 29-item model, each item had a moderate correlation with the subscale to which it belonged. PA negatively correlated with a-SYN (*r* = −0.22***) and AC (*r* = −0.14***) while positively correlating with f-SYN (*r* = 0.11^∗∗^). REV positively correlated with EE (*r* = 0.17***) and AC (*r* = 0.19***). A negligible positive correlation was also found between REV and f-SYN (*r* = 0.08^∗^). f-SYN, a-SYN, and AC were positively correlated with each other.

**TABLE 3:** The correlation between subscales and item-total correlations.

	**1**	**2**	**3**	**4**	**5**	**6**	***M***	***SD***	**Item-total correlation**
PA	(0.67)						2.72	0.70	0.56*** to 0.69***
REV	0.04	(0.71)					2.54	0.70	0.53*** to 0.68***
EE	–0.04	0.17**	(0.79)				3.55	0.83	0.37*** to 0.77***
f-SYN	0.11**	0.08*	0.31***	(0.49***)			3.54	0.80	0.49*** to 0.86***
a-SYN	–0.22***	0.05	0.25***	0.24***	(0.32***)		4.19	0.70	0.75*** to 0.87***
AC	–0.14***	0.19***	0.40***	0.33***	0.38***	(0.75)	3.78	0.56	0.55*** to 0.66***

The Cronbach’s α of subscales are listed in the diagonal position. PA, Passive Acceptance; REV, Revelation; EE, Embeddedness/Emanation; f-SYN, femininity Synthesis; a-SYN, autonomous Synthesis; AC, Active Commitment. ***p < 0.001; **p < 0.01; *p < 0.05.

### Discussion

Study 2 aimed to re-examine the structure validity of the FIC obtained in Study 1. The results of CFAs suggest that the correlated six-factor model is a better fit to the data, which is consistent with our hypothesis. On the one hand, the results of the present investigation indicate that the dimensionality of feminist identity is present in women in mainland China. The structure of four of the five original subscales (except SYN) largely concurs with the findings of Fischer et al. (2000).

On the other hand, the factor structure of the current sample of Chinese women was distinguished by the splitting of the original SYN subscale. More specifically, the SYN subscale was divided into two different but related subscales, a-SYN and f-SYN. As mentioned above, in state-defined equality, i.e., ‘gender sameness,’ femininity is regarded as a factor leading to women’s inferiority (Ji et al., 2017), thus hindering the integration of feminine attributes into a positive self-concept. Although images of feminized women began to flood popular culture only after the 1980s, such resurgence resulted from more stern social pressure after market reform, such as escalating work-family conflicts, growing gender discrimination, and decreasing female employment rates (Ji et al., 2017), rather than the spread of feminist ideologies. In addition, femininity has not always been part of the notion of strong women in feminism. It was not until the occurrence of third-wave feminism and the post-feminist era that femininity began to be emphasized (Nguyen, 2013); specifically, such feminists believe that women deserve to feel like starlets, and queens of sensuality and beauty, in addition to believing that embracing pink things stereotypical of girlhood can be a confident gesture that can contribute to overturning the way society is structured (Nguyen, 2013). Therefore, the splitting of the SYN subscale is explicable.

The correlations between the subscales are partially in line with our expectations. PA was negatively related to a-SYN and AC while positively related to f-SYN, indicating that the different but related structure of a-SYN and f-SYN may have caused the lower psychometric performance of SYN in previous studies and its unclear relationship with the other subscales. In addition, the non-significant relationship of PA with REV and EE suggests that traditional gender ideologies have little to do with the sense of sisterhood in Chinese women.

## Study 3: Convergent Validity

Study 3 aimed to examine the convergent validity of the FIC. Evidence of convergent validity is demonstrated by high correlations with similar measures (Messick, 1989). We examined the correlation of six FIC subscales with willingness to engage in feminist behaviors, and with genderism and transphobia.

Collectivist orientation is an important part of gender consciousness (Gurin et al., 1980). Previous studies have suggested that women who are actively involved in feminist activities prefer to embrace a stronger feminist identity (Conlin and Heesacker, 2018; Frederick and Stewart, 2018). For Chinese women, feminists such as the *Feminist Five*, who have been active in movements, are pioneers in promoting gender equality in China currently. However, due to rigorous censorship, feminists choose to use social media to disseminate information, build a community, and initiate feminist movements to avoid being detained. The Internet has become a new battlefield for Chinese feminism in the gender debate (Huang, 2016). In the present study, we employed the instrument developed by Redford et al. (2016), which taps into individuals’ willingness to engage in feminist behaviors, such as upvoting Internet posts supporting feminist ideas, to examine the relationship between the FIC subscales and feminist behaviors. We hypothesized that (H3a) willingness to engage in feminist behaviors will be positively correlated with the latter five subscales, particularly, EE and AC, both of which are actions or intended actions that reflect feminist attitudes (DeBlaere et al., 2017).

Genderism and transphobia refer to the discrimination against transsexuals, transgenderists, and crossdressers (Hill and Willoughby, 2005). In more recent times, third wave postmodern feminism, and transgender and gender non-conforming (TGNC) were included in feminist issues, and movements toward post-structuralist and queer feminism were launched (Evans, 2015). Platt and Szoka, in press reported the significant inverse correlation between endorsement of feminist beliefs and endorsement of transphobic beliefs in their study. Other researchers have found some factors which are also key aspects of feminism and are thought to be inversely correlated with transphobia (Hill and Willoughby, 2005; Tebbe and Moradi, 2012). Therefore, the present study also uses the relationship of the FIC subscales with genderism and transphobia as evidence of convergent validity. For Chinese women, there is a paucity of specific research examining the relationship between endorsement of feminist beliefs and transphobia. However, prior research has found some indirect evidence of the relationship between feminist beliefs and genderism and transphobia. For example, King et al. (2009) found that the understanding of TGNC individuals increased the support of equal opportunity in Hong Kong Chinese people. Ching and Xu (2018) employed experimental methods with a group of Chinese heterosexual college students to find that gender essentialism, which was criticized by third-wave feminism, leads to more transprejudice. Therefore, it is reasonable to expect that the correlation between feminist beliefs and transphobia also exists in a Chinese sample.

In particular, the present study employed the Genderism and Transphobia Scale (GTS) (Hill and Willoughby, 2005). Winter et al. (2008) examined the GTS with a Hong Kong sample and identified five factors (Anti-Sissy Prejudice, Anti-Trans Violence, Trans Unnaturalness, Trans Immorality, and Background Genderism) with 29 items. While the factor structure in Winter et al. (2008) was appreciably different from that underlying Hill and Willoughby’s (2005) Montreal data, the patterns in gender differences were similar between the two samples. Moreover, transprejudice in Asian samples shares some of the same predictors as in Western samples (Chen and Anderson, 2017). It is hence reasonable to employ the GTS to examine the convergent validity of the FIC for women in mainland China. As mentioned above, we hypothesized (H3b) that higher scores in the latter subscales of the FIC (EE, SYN, and AC) will relate to lower genderism and transphobia, while higher score in PA will relate to higher genderism and transphobia. As REV is more in line with radical feminist ideologies (Szymanski, 2004), which have an ambivalent attitude toward TGNC individuals, we did not make any specific hypotheses regarding it.

### Materials and Methods

#### Measures

##### Feminist Identity Composite

This was the same as in Study 1.

##### Willingness to Engage in Feminist Behaviors

Redford et al. (2016) developed an 11-item Likert-type scale to measure willingness to engage in feminist behaviors, such as ‘Imagine you were reading an Internet post-supporting feminist ideas. How willing would you be to like/up vote the post?’ Participants responded to the items on a scale from (1) *strongly disagree* to (6) *strongly agree*. Higher total scores indicate more willingness to engage in feminist behaviors. Redford et al. (2016) reported that the Cronbach’s alpha of the scale is 0.91. The Cronbach’s alpha of the scale in the present study is 0.88.

##### Genderism and Transphobia Scale

Sexism and attitudes toward trans persons were accessed using the GTS developed by Hill and Willoughby (2005). The GTS is a 32-item Likert scale ranging from (1) *strongly agree* to (7) *strongly disagree*. The GTS is composed of two subscales: transphobia/genderism has 25 items (e.g., ‘Men who cross-dress for sexual pleasure disgust me’) while gender bashing has 7 items (e.g., ‘I have behaved violently toward a woman because she was too masculine’). Higher mean subscale scores indicate less genderism and transphobia. The Cronbach’s alpha of the GTS in Hill and Willoughby’s (2005) study was 0.95 for the transphobia/genderism subscale; 0.87 for the Gender bashing subscale; and 0.96 for the total score. The construct validity and Cronbach’s alpha of the GTS was examined before the following analysis.

#### Procedure

Data collection occurred during December 2018. The ethics research committee of Southwest University approved this study. The participants in Study 3 were recruited using the same Web-based survey methods as in Study 1.

#### Participants

We obtained 653 completed questionnaires. For the purpose of this study, we excluded juveniles (age < 18; *n* = 4). The final sample consisted of 649 Chinese women from 25 provinces/regions of mainland China. The age of the sample ranged from 18 to 69 with a mean of 27.24 years (*SD* = 7.00). Most respondents had a full-time job (*n* = 451, 69.5%), while a small number of respondents were full-time students (*n* = 151, 23.3%), and fewer were employed part-time (*n* = 42, 6.5%) or unemployed (*n* = 5, 0.8%). Regarding education, 487 (75.0%) had a college education, 58 (8.9%) had a postgraduate education or higher, 120 (14.8%) had a junior college education, and 82 (12.6%) had high school education or less. Concerning monthly salary, 209 (32.2%) participants earned 3,000–6,000 RMB per month, 170 respondents earned 6,000–10,000 RMB per month (26.2%), 194 respondents earned less than 3,000 RMB per month (29.9%), and 76 earned more than 10,000 RMB (11.7%).

### Results

The internal consistency of the revised FIC in the sample in Study 3 was examined. The Cronbach’s alphas of subscales were 0.68 for PA, 0.75 for REV, 0.81 for EE, and 0.73 for AC. The correlation between the items in f-SYN was 0.56 (*p* < 0.001) and that in a-SYN was 0.27 (*p* < 0.001). The Cronbach’s alpha of the overall revised FIC was 0.73. CFA was conducted with the sample in Study 3. The model fit index met the conventionally accepted cut-offs: χ^2^(362) = 790.037, *p* < 0.001; CFI = 0.90; SRMR = 0.054; RMSEA = 0.043, 90% CI = [0.039, 0.047].

#### Construct Validity of the GTS

We first examined the construct validity of Winter et al.’s (2008) model with the present sample, and the data did not fit the model well (χ^2^[340] = 1765.292, *p* < 0.001, CFI = 0.83, SRMR = 0.063, RMSEA = 0.080, 90% CI = [0.076, 0.084]). For evaluating the construct validity of the GTS with the current sample (*n* = 649), EFA was conducted. The following five factors emerged: Anti-Sissy Prejudice (ASP)—antipathy toward gender-variant men; Anti-Trans Violence (ATV)—a violent antipathy extending to cross-gendered behavior in both sexes; Anti-Tomboy Prejudice (ATP)—antipathy toward gender-variant women; Gender Flexibility (GF)—accepting of unconventional gender expression; and Trans Unnaturalness (TU)—certain beliefs about the nature of gender variance, specifically, the extent to which it violates either a divine or natural order. The five factors totally accounted for 47.41% of the total extracted variance. CFA was then conducted, and the model fit of the data was deemed well: χ^2^(340) = 1043.782, *p* < 0.001, CFI = 0.91, SRMR = 0.043, RMSEA = 0.056, 90% CI = [0.052, 0.060]. The Cronbach’s alpha of the GTS was 0.87 for ASP, 0.77 for ATV, 0.82 for ATP, 0.63 for GF, and 0.84 for TU.

#### Convergent Validity of the FIC

With regard to convergent validity, willingness to engage in feminist behaviors was positively correlated with REV (*r* = 0.24***), EE (*r* = 0.31***), f-SYN (*r* = 0.15***), a-SYN (*r* = 0.25***). AC yielded the strongest correlation with (*r* = 0.53***) willingness to engage in feminist behaviors.

With regard to the relationship between the FIC and GTS, PA was negatively correlated with all the subscales of the GTS (Anti-Sissy Prejudice, *r* = −0.39***; Anti-Trans Violence, *r* = −0.28***; Anti-Tomboy Prejudice, *r* = −0.41***; Gender Flexibility, *r* = −0.38***; and Trans Unnaturalness, *r* = −0.41***). REV negatively correlated with Anti-Trans Violence (*r* = −0.11**). f-SYN also yielded significantly negative correlations with the five factors of the GTS (Anti-Sissy Prejudice, *r* = −0.11**; Anti-Trans Violence, *r* = −0.14***; Anti-Tomboy Prejudice, *r* = −0.14***; Gender Flexibility, *r* = −0.13***; Trans Unnaturalness, *r* = −0.17***). a-SYN yielded a significantly positive correlation with Anti-Tomboy Prejudice (*r* = 0.10*) and Gender Flexibility (*r* = 0.10*), and a negligible positive correlative with Anti-Sissy Prejudice (*r* = 0.09*). AC was positively correlated with Gender Flexibility (*r* = 0.14**). The results are shown in Table 4.

**TABLE 4:** Descriptive and bivariate correlations for the total sample.

	**1**	**2**	**3**	**4**	**5**	**6**	**7**	**8**	**9**	**10**	**11**	**12**	***M***	***SD***
PA	(0.68)												2.66	0.68
REV	0.03	(0.75)											2.42	0.69
EE	–0.01	0.24***	(0.81)										3.63	0.80
f-SYN	0.16***	0.06	0.22***	(0.56***)									3.59	0.82
a-SYN	–0.15***	0.10*	0.25***	0.38***	(0.27***)								4.12	0.69
AC	–0.14***	0.25***	0.38***	0.31***	0.37***	(0.73)							3.75	0.54
WEFB	–0.15***	0.24***	0.30***	0.14***	0.24***	0.53***	(0.88)						4.35	0.78
ASP	–0.40***	0.08*	0.04	–0.11**	0.09*	0.09*	0.16***	(0.87)					4.16	1.31
ATV	–0.28***	–0.11**	–0.04	–0.14***	–0.03	–0.03	–0.02	0.62***	(0.77)				5.63	0.99
ATP	–0.41***	–0.06	–0.07	–0.14***	0.10*	0.03	0.09*	0.64***	0.62***	(0.82)			5.64	1.14
GF	–0.38***	0.03	–0.03	–0.13***	0.10*	0.14**	0.13**	0.65***	0.49***	0.60***	(0.63)		4.93	1.05
TU	–0.41***	0.05	–0.00	–0.17***	0.06	0.06	0.07	0.66***	0.42***	0.50***	0.56***	(0.84)	4.20	1.86

The Cronbach’s α of scales are listed in the diagonal position. PA, Passive Acceptance; REV, Revelation; EE, Embeddedness/Emanation; f-SYN, femininity Synthesis; a-SYN, autonomous Synthesis; AC, Active Commitment; WEFB, willingness to engage in feminist behavior; ASP, Anti-Sissy Prejudice; ATV, Anti-Trans Violence; ATP, Anti-Tomboy Prejudice; GF, Gender Flexibility; TU, Trans Unnaturalness. ***p < 0.001; **p < 0.01; *p < 0.05.

### Discussion

As hypothesized, AC—a manifestation of feminist awareness—yielded the strongest correlation with willingness to engage in feminist behaviors, indicating that women who intend to participate in the feminist movement also prefer to engage in daily feminist behaviors. The positive correlation between the four latter subscales with willingness to engage in feminist behaviors provides evidence for the convergent validity of the FIC.

Consistent with our hypotheses, the significant negative correlations between PA and all the subscales of the GTS indicate that women with stronger traditional values exhibit more transprejudice. The hypothesis referring to the positive correlation between the FIC’s latter five subscales with the GTS is partially supported. Of the 25 possible correlations between the latter five FIC subscales and the GTS, 12 were significant and in the expected directions (e.g., the positive correlation of a-SYN with ATP and GF, and between AC and ATP). These results suggest that the role of feminism in attitude toward TGNC individuals is fraught with controversy in China. In addition, the negative correlations of f-SYN with all the subscales of the GTS also indicate that the appreciation of femininity is more in line with traditional gender ideologies rather than feminist beliefs for Chinese women.

## General Discussion

This research presents three studies aimed at examining the psychometric properties and underlying factor structure of the FIC using EFA and CFA procedures with different samples of women in mainland China. In Study 1, EFA using the item scores obtained from the scale construction sample suggested a correlated six-factor structure for the FIC. Unlike Fischer et al. (2000), two distinct forms of Synthesis were derived from the original subscale. The new subscales were each composed of two items. In Study 2, the structure validity of the model in Study 1 was examined using CFA with a different sample, and the model fit index met the conventionally accepted cut-offs. In Study 3, the convergent validity was examined by employing the correlations between the FIC subscales and discrimination measures, and the feminist engagement. Finally, the results provide some evidence for the cross-cultural validity of the FIC for women in mainland China.

### Underlying Factor Structure of the FIC in Women in Mainland China

After performing EFA and CFA with different samples, the factor structure reported by Fischer et al. (2000) was generally supported, indicating that elements of feminist identity also exist in Chinese women. Consistent with previous studies (Moradi and Subich, 2002; DeBlaere et al., 2017), the correlations between the six subscales of the FIC reaffirm that it is more appropriate to consider the stages as different feminist ideologies rather than a sequential or linear model of feminist identity.

However, the factor structure obtained with the present sample of women in mainland China is distinguished by two aspects. First, the original SYN subscale was split into two subscales: f-SYN and a-SYN. As per the above discussed reasons for the split, it is imperative to determine the role of femininity and autonomy in feminist identity. It is well-understood that the image of independent and competent women is encouraged and pursued by feminism (Perkins and Schreiber, 2019). However, different feminist groups endow femininity with distinctive values (Cole and Zucker, 2007). For example, second-wave feminists regard femininity as a set of embodied characteristics and practices imposed on women which result from or signify their subordinate status in relation to men (Schippers and Sapp, 2012), while for third-wave feminists, femininity is embodied as a surface performance available to everyone, regardless of sex category (Schippers and Sapp, 2012). Third-wave feminists encourage ‘girls’ to immerse themselves in the pleasures of femaleness (Lane et al., 2016; Siegel and Calogero, 2019). Therefore, women whose views are more in line with second-wave feminists would reject femininity and get an inverse score in two subscales of SYN, while supporters of third-wave feminism would not. In addition, women’s understanding of the connotation of femininity also affects their SYN scores. In particular, for Chinese women, femininity is also emphasized in traditional gender ideology. We call this the patriarchal construct of femininity, the connotation of which is obedience, domesticity, sexual fidelity, and sexlessness, while the feminist construct of femininity means bodily practice, and qualities of starlets and queens of sensuality and beauty (Nguyen, 2013). People’s support for femininity depends on their definition of femininity. We can speculate that a traditional woman who regards the concept of ‘feminine’ in the f-SYN items as a patriarchal construct would also obtain a higher score in f-SYN, while a feminist who defines ‘feminine’ in the same way would strongly disagree with the items in f-SYN. These results seem to indicate that such controversial issues need to be clarified by researchers when assessing people’s feminist beliefs.

Second, the relationships between the FIC subscales also yielded a distinctive pattern from that found in previous studies (Fischer et al., 2000; Yoder et al., 2012; DeBlaere et al., 2017). For example, the negative relationship between EE and PA exhibited in previous studies was not found among Chinese women. As Frederick and Stewart (2018) suggested, the development of a feminist identity does not always occur in the same order. Furthermore, all the stages are not always applicable to the process by which women acquire a feminist identity. The heterogeneity of the process results from some contextual factors, such as feminist education, social relationships, and experience with gender-based prejudice (Frederick and Stewart, 2018). Therefore, the particular pattern of relationships between the subscales may indicate the unique process by which women obtain a feminist identity.

### Convergent Validity

The convergent validity of the FIC is completely supported by the correlation of the six subscales with willingness to engage in feminist behaviors and partially supported by the correlations between the subscales of the FIC with the GTS. As hypothesized, willingness to engage in feminist behaviors positively correlated with the latter stages of feminist identity development. In particular, AC had the highest magnitude correlations with willingness to engage in feminist behaviors, given that AC is the manifestation of feminist awareness (DeBlaere et al., 2017).

As reported earlier, attitude toward TGNC individuals was not closely related to the dimensions of feminist identity development, which can be explained from the following aspects. First, there has been a longstanding debate regarding whether TGNC individuals and LGBT issues should be included in feminist movements (De Oliveira et al., 2011). Some feminists, particularly the pioneers of second-wave feminism, believe that TGNC individuals should be explicitly excluded (Browne, 2011). Therefore, women with a strong feminist identity may not necessarily have lower genderism and transphobia. In addition, although people with feminist values may have less prejudice toward TGNC individuals, such prejudice is also affected by other specific individual-level factors, such as openness to experience (Cramer et al., 2013), as well as socio-demographic features, such as educational experiences, gender, and religion (Scandurra et al., 2017). Due to the effect of these diverse factors, the relationship between feminist beliefs and transprejudice is quite complex. In the present research, a strong feminist identity did not yield a significant relationship with all kinds of transphobia (only AC was positively related to GF, and a-SYN was positively related to ATP and GF). In addition to the abovementioned reasons, we should also consider the obstacles related to trans issues embedded in Chinese society. TGNC individuals are still often marginalized in the Chinese feminist movement (Ye, 2016). Only some transgender celebrities have caught the attention of feminists. However, such biased information blinds people to the real-life struggles of trans people (Ye, 2016). Therefore, the relationship between TGNC individuals and Chinese feminism needs to be further examined.

### Limitations

Several limitations should be acknowledged in the present study. First, the revised model has two defects. On the one hand, the internal consistency reliability of a-SYN did not meet the acceptable cut-off. On the other hand, the two subscales of SYN contained only two items, which does not meet the factor retention standard of scale development (Tabachnick and Fidell, 2007). Both of them limited the employment of the FIC to measure Chinese women’s feminist identity. In addition, genderism and transphobia measures did not strongly support the convergent validity of the FIC. We suggest that future studies re-examine the validity of the FIC with distinct measures, which could also give significance to developing a measure of Chinese women’s feminist identification that emerges from within Chinese culture itself.

Second, the use of the Internet for data collection limits the ability to generalize findings to larger populations. The website we used in the current study is similar to Amazon’s Mechanical Turk (MTurk), which helps scholars efficiently collect large-scale and diverse data (Paolacci and Chandler, 2014). Web-based data collection may nonetheless exclude women who have difficulty accessing the Internet or who are unfamiliar with the website. Future studies should employ different means of data collection to explore whether the factor of the FIC found in the current study can be replicated among women with different characteristics in China.

### Practice Implications

By examining the psychometric properties of the FIC with sample of women in mainland China, we obtained the some evidence of cross-cultural validity of the FIC, particular in Asian cultural background, which give implication for the measurement’s future employment and possible amendment.

First, the Synthesis scale may need urgent revision. As Synthesis had unsatisfactory reliability performance in the original FIC (Erchull et al., 2009), the invalidity of Synthesis may be magnified under the non-Western cultural background. Particularly, the perception of femininity need to be articulated in the FIC. Second, are traditional gender attitudes measured by PA still suitable for modern society? As discrimination against women has changed overtime (Henley et al., 1998) and become increasingly subtle and covert (Swim et al., 1995), old-fashioned and modern sexism have become distinct from one another (Swim et al., 1995). As previous studies developed different instruments to measure the traditional and egalitarian beliefs (Yoon et al., 2015), we suggest that the applicability of Passive Acceptance to modern society must be re-examined.

The findings in the present study, to our knowledge, are the first quantitative evidence for feminist identity in Chinese women, and we hope that it will draw more scholars’ attention to the discrepancy between feminism in Western countries and non-Western countries, particularly China. Although the feminist activities in China are increasingly similar to the West, the cultural factors and development course make things different (Dong, 2005; Ni, 2005). For example, the Western framework which pitched women against men in their critique of traditional social-science epistemology or in their analysis of social injustice has not been well-received in Asian societies (Cheung, 2012). In addition, general support for online feminist behaviors in the present study also suggests that feminism in China must be achieved in a quieter way than in Western countries. Under strict censorship, feminist also require a balanced route ‘without challenging the false universality of male desires and abilities’ (Lin et al., 1998) if they are to practice feminist ideology (Liu and Dahling, 2016). Consequently, the Internet and cyberspace may be the main battlefield for Chinese feminism.

## Data Availability Statement

The datasets analyzed in this manuscript are not publicly available. Requests to access the datasets should be directed to YL, lyj0214@email.swu.edu.cn.

## Ethics Statement

The study was carried out in accordance with the recommendations of the Ethics Committee of Southwest University with written informed consent from all participants before the surveys. All participants gave written informed consent in accordance with the Declaration of Helsinki. The protocol was approved by the Ethics Committee of Southwest University.

## Author Contributions

Both authors contributed equally to the design of the study. YL executed the study, conducted the data analyses, and wrote the manuscript. YZ advised on the execution and analyses of the study, and collaborated in the editing of the manuscript.

## Conflict of Interest

The authors declare that the research was conducted in the absence of any commercial or financial relationships that could be construed as a potential conflict of interest.

## Footnotes

1 https://www.wjx.cn/

2 https://www.wjx.cn/
