# Supplementary material for: Reliability and Validity of Feminist Identity Composite in Chinese Women
Source: Front Psychol. 2019 Dec 20;10:2842. doi: 10.3389/fpsyg.2019.02842 (PMC6932982; doi:10.3389/fpsyg.2019.02842)
Supplement: Supplementary file 1 [file Table_1.DOCX]

Supplementary Material

# 1. Feminist Identity Composite

| **No.** | **Items** |
| --- | --- |
| PA1 | 与男人不同，女人不会被期待去做一些例如建筑工程、赛车等危险的工作，这点我觉得很幸运 |
|  | I think it’s lucky that women aren’t expected to do some of the more dangerous jobs that men are expected to do, like construction work or race car driving |
| PA2 | 我认为过去那种男人挣钱，女人顾家的状态更好 |
|  | I think that men and women had it better in the 1950s when married women were housewives and their husbands supported them |
| PA4 | 我喜欢做一个传统的女性 |
|  | I like being a traditional female |
| PA5 | 作为一个女人，我特别喜欢的一件事是，男人会因为我是一个女人而在拥挤的公共汽车上给我让座或者为我开门 |
|  | One thing I especially like about being a woman is that men will offer me their seat on a crowded bus or open doors for me because I am a woman |
| PA6 | 如果我嫁给了一个男人，而他却在另一个省份找到了一份工作，那么我有义务随他搬家以支持他的事业 |
|  | If I were married to a man and my husband was offered a job in another state, it would be my obligation to move in support of his career. |
| PA7 | 我认为，大多数女性会因为成为一名妻子和母亲而感到最大的满足 |
|  | I think that most women will feel most fulfilled by being a wife and a mother. |
| REV1 | 我觉得自己被欺骗了而认同社会对我作为一个女性的看法 |
|  | I feel like I’ve been duped into believing society’s perceptions of me as a woman. |
| REV2 | 直到最近我才意识到我作为一个女人在社会中经历过压迫和歧视 |
|  | I never realized until recently that I have experienced oppression and discrimination as a woman in society |
| REV4 | 我可以意识到过去的我是如何坚定地认同着性别歧视，这一点令我后悔 |
|  | Regretfully, I can see ways in which I have perpetuated sexist attitudes in the past |
| REV5 | 在我与男人的交往中，我总是寻找由于我是女人而可能被歧视的方式 |
|  | In my interactions with men, I am always looking for ways I may be discriminated against because I am female. |
| REV6 | 我的女性朋友就像我一样，我们都对男人感到愤怒，也对我们由于是女人而受到的待遇感到愤怒 |
|  | My female friends are like me in that we are all angry at men and the ways we have been treated as women |
| REV7 | 每当想到男人或者男孩对待我的方式，我就会感到生气 |
|  | I feel angry when I think about the way I am treated by men and boys |
| REV8 | 男人会在社会上享有很多优势，他们因此反对与女性平等共处 |
|  | Men receive many advantages in society and because of this are against equality for women |
| EE1 | 我对女性音乐家非常感兴趣 |
|  | I am very interested in women musicians. |
| EE2 | 我对女性作家非常感兴趣 |
|  | I am very interested in women writers. |
| EE3 | 我对女性艺术家非常感兴趣 |
|  | I am very interested in women artists. |
| EE4 | 我对有关女性的研究非常感兴趣 |
|  | I am very interested in women’s studies. |
| SYN1 | 我感觉我已经把我的女性特质和独特的个人品质融为一体了 |
|  | I feel like I have blended my female attributes with my unique personal qualities |
| SYN2 | 我已经把“女性”和“女性气质”融入进我自己独特的人格特征中 |
|  | I have incorporated what is female and feminine into my own unique personality |
| SYN3 | 我为成为一个能干的女人而感到自豪 |
|  | I am proud to be a competent woman. |
| SYN4 | 我享受着因成为一名强大的女性而获得的骄傲与自信 |
|  | I enjoy the pride and self-assurance that comes from being a strong female |
| AC1 | 我非常致力于此（女性主义）的原因是，我相信它将有助于为所有人创造一个更加公平和公正的世界 |
|  | I am very committed to a cause that I believe contributes to a more fair and just world for all people. |
| AC2 | 我非常关心男女在各方面都能享有平等的机会 |
|  | I care very deeply about men and women having equal opportunities in all respects. |
| AC3 | 我愿意为所有人做出一些牺牲来有效地改变这个社会和这个世界，以创造一个无性别歧视、人人都有平等机会的和平之地 |
|  | I am willing to make certain sacrifices to effect change in this society in and just world for all people. Order to create a nonsexist, peaceful place where all people have equal opportunities. |
| AC4 | 从某种程度上说，我参与的几乎每一个活动的动机都出于我对平等主义世界的渴望 |
|  | On some level, my motivation for almost every activity I engage in is my desire for an egalitarian world. |
| AC5 | 我想努力提高妇女的社会地位 |
|  | I want to work to improve women’s status. |
| AC6 | 我认真地选择我的“事业”，并以此努力为所有人争取更大的平等 |
|  | I choose my “causes” carefully to work for greater equality of all people. |
| AC7 | 对于能在女性运动中发挥我的才能和技能，我感到非常满足 |
|  | It is very satisfying to me to be able to use my talents and skills in my work in the women’s movement. |
| AC9 | 对于我现在关注的妇女问题，我是一个有力且实际的发言人 |
|  | I feel that I am a very powerful and effective spokesperson for the women’s issues I am concerned with right now |

# 2. Genderism and Transphobia Scale.

| **Item** | **Subscale** |
| --- | --- |
|  | **Anti Sissy Prejudice** |
| 22 | If a man wearing make-up and a dress, who also spoke in a high voice, approached my child, I would use physical force to stop him. |
|  | 我会用身体力量去阻止一个化妆、穿裙子，声音尖细的男人靠近我的孩子 |
| 25 | Feminine men make me feel uncomfortable. |
|  | 阴柔的男人另我不舒服 |
| 17 | A man who dresses as a woman is a pervert. |
|  | 穿着打扮像女性一样的男人是个变态 |
| 7 | Men who cross-dress for sexual pleasure disgust me. |
|  | 为了性快感而穿异性服装的男人让我恶心 |
| 21 | Passive men are weak. |
|  | 消极的男人是软弱的 |
| 26 | I would go to a bar that was frequented by females who used to be males. |
|  | 我愿意去一个变性女人（原本是男人）常去的酒吧 |
| 19 | Feminine boys should be cured of their problem. |
|  | 表现阴柔的男人应该就此问题进行相应的治疗 |
| 3 | If I found out that my best friend was changing their sex, I would freak out. |
|  | 如果我发现我最好的朋友正准备改变自己的性别，我会抓狂的 |
| 10 | Men who act like women should be ashamed of themselves. |
|  | 行为表现如女人一般的男人应该为自己感到羞耻 |
| 16 | I would avoid talking to a woman if I knew she had a surgically created penis and testicles. |
|  | 如果我知道一个女人有一个手术制造的阴茎和睾丸，我会避免和她交谈 |
|  | **Anti-Trans Violence** |
| 28 | My friends and I have often joked about men who dress like women. |
|  | 我和我的朋友经常拿穿得像女人的男人开玩笑 |
| 20 | I have behaved violently toward a man because he was too feminine. |
|  | 我曾因为一个男人太女性化而对他表现粗暴 |
| 6 | I have teased a man because of his feminine appearance or behavior. |
|  | 我曾因为一个男人阴柔的形象或行为而取笑他 |
| 1 | I have beat up men who act like sissies. |
|  | 我打过那些像娘娘腔一样的人 |
| 2 | I have behaved violently toward a woman because she was too masculine. |
|  | 我曾因为一个女性太男性化而对她表现粗暴 |
| 18 | If I found out that my lover was the other sex, I would get violent. |
|  | 如果当发现我的爱人/恋人是变性者，我将会对他/她变得暴力 |
| 31 | It is all right to make fun of people who cross-dress. |
|  | 取笑那些穿异性服装的人是没关系的 |
|  | **Anti-Tomboy Prejudice** |
| 29 | Masculine women make me feel uncomfortable. |
|  | 阳刚的女人令我不舒服 |
| 15 | Women who see themselves as men are abnormal. |
|  | 把自己当作男人的女人是不正常的 |
| 30 | It is morally wrong for a woman to present herself as a man in public. |
|  | 对女人来说，在公共场合以男人的身份表现自己是不道德的 |
| 12 | I cannot understand why a woman would act masculine. |
|  | 我无法理解为什么一个女人会表现出男性化 |
|  | **Fluid Gender Expression** |
| 14 | Children should play with toys appropriate to their own sex. |
|  | 孩子们应该玩跟自己性别相匹配的玩具 |
| 9 | If I saw a man on the street that I thought was really a woman, I would ask him if he was a man or a woman |
|  | 如果我无法准确地判断一个人到底是男性还是女性，那么我会尊重他对自己性别的选择 |
| 23 | Individuals should be allowed to express their gender freely. |
|  | 应该允许个人自由地表现他们的性别 |
| 5 | If a friend wanted to have his penis removed in order to become a woman, I would openly support him. |
|  | 如果一个朋友为了成为一个女人而想要把他的阴茎切除，我会公开支持他 |
| 8 | Children should be encouraged to explore their masculinity and femininity. |
|  | 我们应该鼓励孩子既探索他/她们的男性气质和也探索他/她们的女性气质 |
|  | **Trans Unnaturalness** |
| 4 | God made two sexes and two sexes only. |
|  | 上帝创造了两种性别，也只能有这两种性别 |
| 27 | People are either men or women |
|  | 人类，不是男人就是女人 |
